# Reliability of Endoscopic Ultrasound Using Miniprobes and Grayscale Histogram Analysis in Diagnosing Upper Gastrointestinal Subepithelial Lesions

**DOI:** 10.1155/2020/6591341

**Published:** 2020-06-09

**Authors:** Samiullah Khan, Rui Zhang, Weili Fang, Tao Wang, Shu Li, Danna Wang, Yixiang Chang, Lanping Zhu, Bang-mao Wang, Wentian Liu

**Affiliations:** Department of Gastroenterology and Hepatology, Tianjin Medical University General Hospital, Tianjin., China

## Abstract

**Background:**

To assess the role of endoscopic ultrasound (EUS) in the diagnosis of upper gastrointestinal subepithelial lesions (SELs) and to investigate EUS combined with a grayscale histogram analysis for the differentiation of leiomyomas and gastrointestinal stromal tumors (GISTs).

**Methods:**

A retrospective study of 709 patients with upper gastrointestinal SELs was conducted by EUS before endoscopic resection. The EUS findings of SELs and pathological results after endoscopic resection were compared. The EUS images of SELs, particularly, leiomyoma and GIST, were further analyzed via a grayscale histogram to differentiate between the two tumors.

**Results:**

Of the 709 patients, 47 cases were pathologically undetermined. The diagnostic consistency of EUS with endoscopic resection was 88.2% (584/662), including 185 muscularis mucosa, 61 submucosa, and 338 muscularis propria, respectively. The diagnostic consistency of EUS with pathology was 80.1% (530/662). The gray value of GISTs was significantly higher than that of leiomyomas (58.9 ± 8.3 vs. 39.5 ± 5.9, *t* = 57.0, *P* < 0.0001). The standard deviation of leiomyomas was significantly lower than that of GISTs (20.6 ± 7.0 vs. 39.8 ± 9.3, *t* = 23.7, *P* < 0.0001). The grayscale histogram analysis of GISTs showed higher echo ultrasound, and the echo of leiomyoma was more uniform.

**Conclusion:**

EUS is the preferred procedure for the evaluation of upper gastrointestinal SELs. EUS combined with a grayscale histogram analysis is an effective method for the differentiation of leiomyomas and GISTs.

## 1. Introduction

Subepithelial lesions (SELs) are tumors covered by normal-appearing mucosa and usually found incidentally during routine upper gastrointestinal endoscopy. These tumors originate from the muscularis mucosa, submucosa, or muscularis propria. They occur more frequently in the stomach, esophagus, and duodenum, such as gastrointestinal stromal tumor (GIST), leiomyoma, neuroendocrine tumor, granular cell tumors, lipoma, ectopic pancreas, and schwannoma. Most subepithelial lesions are benign at the time of diagnosis, with less than 15% found to be malignant at presentation. However, many of these lesions have the potential for malignant transformation [1–3]. There is a broad differential diagnosis of such lesions, which emphasizes the importance of an accurate diagnosis. Lesions that are usually identified by routine endoscopy but not properly diagnosed often require computed tomography (CT). The accuracy of CT can be difficult to determine, especially for tumors that are too small or located within the gastrointestinal tract wall [4].

Besides, routine endoscopic biopsies and collecting tissue samples for diagnosis can be difficult, as SELs are located beneath the normal epithelial layer. However, endoscopic ultrasound (EUS) allows the practitioner to extract tissue samples since endoscopic ultrasound-guided fine-needle aspiration (EUS-FNA), and the EUS-guided fine needle biopsy (EUS-FNB) is an investigation that allows tissue acquisition before endoscopic resection [2, 5]. With regard to specific clinical indications, the role of EUS and EUS-FNA in the localization staging of luminal gastrointestinal cancers is well known [6]. Due to its high sensitivity and specificity, endoscopic ultrasound has been recognized as an accurate imaging method for the evaluation of SELs in the gastrointestinal tract [1, 2, 7–10]. Echo intensity, homogeneity, and tissue or other material characteristics that reflect ultrasound waves are typically used as diagnostic sonographic standards [11]. Nevertheless, the main challenge is to differentiate between leiomyomas and GISTs. Manual assessment causes a significant subjective error and, for this reason, the grayscale histogram analysis is a widely cited method. It has been widely used for a variety of image editing programs. The grayscale histogram analysis has been extensively used for ultrasound imaging in processing muscle quantification [12], quantifying cerebral hemorrhage [13], and immunohistochemistry [14]. The purpose of this study was to evaluate the importance of EUS in the diagnosis of upper gastrointestinal SELs and to explore further use of EUS in conjunction with the grayscale histogram analysis to distinguish between leiomyoma and GIST in a fairly large number of cases in a single center.

## 2. Materials and Methods

### 2.1. Patients

This was a retrospective study to assess the reliability and efficacy of EUS and EUS combined with the grayscale histogram analysis for the diagnosis of SELs in the upper gastrointestinal tract. Between April 2010 and March 2018, 709 consecutive patients (282 male and 427 female) with upper gastrointestinal SELs were examined by EUS before endoscopic resection. Of the 709 patients, 47 cases (mean age and tumor size, 50.2 ± 11.1, 2.0 ± 0.8) were pathologically undetermined. However, the mean age of patients with GIST and leiomyoma showed 57.1 ± 9.8 vs. 52.8 ± 10.9, ectopic pancreas (50.3 ± 11.2), and lipoma (50.1 ± 12.9), respectively (Table 1).

### 2.2. Methods

All EUS examinations were performed by two experienced endosonographers (W.L. and B.M.W.) using an endoscope (CV-260SL, Olympus, Tokyo, Japan) and an ultrasound miniprobe (UM-DP20-25R, frequency 20 MHz) to evaluate SELs. The setting of the gain level and contrast level in the EUS apparatus was adjusted as (G:15/C:5). The mucus and foam in the upper digestive tract have been absorbed, washed, and sucked several times with diluted simethicone. The gas in the lumen was adjusted by suction after lesion exposure, and sterile water was used for filling. In addition, patients were changed in position when needed, so that the water could cover the lesion and used as a medium for ultrasound examination to explore the tumor origin layer, the echo level and uniformity, size, boundary, the direction of tumor growth, and the relationship with surrounding organs.

All cases underwent endoscopic resection with endoscopic mucosal resection (EMR), endoscopic trepanned resection (ETR), endoscopic submucosal dissection (ESD), submucosal tunneling endoscopic resection (STER), and/or endoscopic full-thickness resection (EFTR). After endoscopic resection, all resected tissues were immediately fixed in 10% neutral formalin and routinely embedded for histological examination. Immunohistochemistry analyses for CD117, CD34, smooth muscle actin, desmin, S-100, and DOG-1 were performed to determine pathological diagnosis. The type of lesions, postoperative pathology, and immunohistochemical results were compared to determine the EUS lesion type. The diagnosis was considered to be reliable when both results showed identical findings. Furthermore, EUS imaging of SELs, in particular, leiomyoma and GIST, were further analyzed using a grayscale histogram for tumor tissue echogenicity by measuring the mean gray value and the mean gray value of the standard deviation to differentiate between the two tumors.

### 2.3. Grayscale Histogram Analysis

A grayscale histogram-based EUS imaging analysis was performed to measure changes in the gray value. To calculate and display a histogram for the distribution of gray values in the active image, the region of interest manager is a tool that works with multiple selections from different locations in an image. According to Harris-Love et al. [12], the Rectangular Marquee Tool and the FreeHand Tool are two types of editing features used to perform a grayscale histogram analysis to determine the region of interest within an ultrasound image. In this study, the region of interest in ultrasound images was further defined by an experienced endosonographer (W.L.). Regions with the most hypoechoic areas with adjacent tissues have been selected as regions of interest. Furthermore, to reduce the error, each image was measured five times and the data were statistically analyzed.

**Figure 1:**
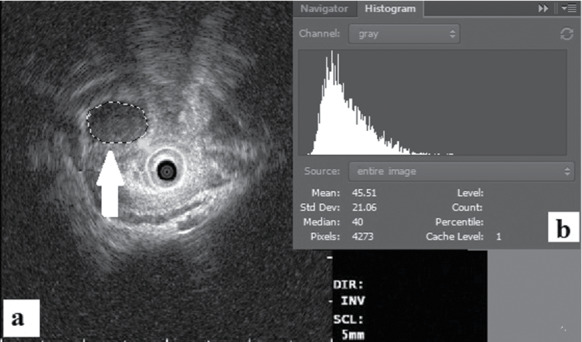
Endoscopic ultrasound combined with grayscale histogram (a, b).

The mean gray value within the selection is the sum of gray values of all the pixels in the selection. The standard deviation of the gray value used to generate the mean gray value. Median refers to the median value of the pixels in the image or in the selection. Lasso tool was performed for the area selection of tumors to measure the mean gray value and the mean gray value of the standard deviation (Figure 1). The main observation was the gray value of lesions. The mean gray value represents the intensity of the echo, and the mean gray value of the standard deviation represents the uniformity. The EUS images of both leiomyoma and GIST consistent with the pathological diagnosis were processed via a grayscale histogram analysis in order to verify whether the abovementioned method is feasible.

### 2.4. Statistical Analysis

All statistical analyses were performed using SPSS (version 18.0). Baseline data are presented as mean ± SD. The *t*-test was used for testing the significance between quantitative variables using unit record data. The level of statistical significance was set at two-tailed *P* < 0.05.

### 2.5. Patient and Public Involvement

This was a retrospective study; neither patients nor the public was involved in this study.

### 2.6. Ethics Statement

This study was approved by the ethical review board of Tianjin Medical University General Hospital (reference no: IRB2015-YX-009).

## 3. Results

### 3.1. Diagnostic Consistency of EUS

Of the 662 patients diagnosed with EUS before endoscopic resection, EUS was very effective in the clinical assessment of SELs, including GIST (*n* = 281), leiomyoma (*n* = 317), ectopic pancreas (*n* = 31), and lipoma (*n* = 33). The mean tumor size (cm) of GIST and leiomyoma showed 1.5 ± 0.9 vs. 1.2 ± 0.6, ectopic pancreas (1.9 ± 0.9), and lipoma (2.1 ± 0.9), respectively. Subepithelial lesions found in the upper gastrointestinal tract, including the esophagus (279 cases), stomach (378 cases), and duodenum (5 cases). Moreover, leiomyomas and GISTs were the most common tumors found in the stomach (Table 2).

The diagnostic consistency of EUS with endoscopic resection was 88.2% (584/662). The number of tumors originated from muscularis mucosa 76.4% (185/242), submucosa 88.4% (61/69), and muscularis propria 96.6% (338/350), respectively (Table 3). However, in one case, the origin of the lesion was unclear, and during endoscopic resection, the lesion was found to be diffused and penetrated the muscular propria.

Furthermore, the diagnostic consistency of EUS with postoperative pathology showed 80.1% (530/662), GIST 63.0% (177/281), leiomyoma 91.8% (291/317), ectopic pancreas 96.8% (30/31), and lipoma 97.0% (32/33), respectively (Table 4).

### 3.2. Diagnostic Yield

Although upper gastrointestinal SELs are easier to discover by routine endoscopic examination, it is difficult to diagnose the origin and nature of the lesions. EUS is currently the most reliable diagnostic method for distinguishing between intramural lesions and extraluminal compressions and plays a crucial role in the diagnosis and management of SELs. It also demonstrates a hierarchical structure of the upper gastrointestinal wall, adjacent organs and tissues, the layer of origin and echo patterns of SELs, and the precise sonographic characteristics of the lesion. However, the EUS should not be used as the ultimate diagnostic method, and the diagnosis of the lesion needs to be combined with pathological results. Both leiomyoma and GIST are hypoechoic lesions which require histological and immunohistochemical tissue sampling to determine pathological diagnosis [15]. In this study, a grayscale analysis was conducted for pathologically determined leiomyomas and GISTs in order to distinguish between the two tumors.

### 3.3. Comparison of Leiomyoma and GIST Using Grayscale Histogram Analysis

The mean gray value and the mean gray value of the standard deviation between leiomyoma and GIST were significantly different. A grayscale value of 45 was set for the mean gray value, and a grayscale value of 30 was set for the gray value standard deviation to discriminate leiomyoma and GIST. The mean gray value of GISTs was significantly higher than that of leiomyomas (58.9 ± 8.3 vs. 39.5 ± 5.9, *t* = 57.0, *P* < 0.0001), indicating that the intensity of the echo of GISTs was higher than that of leiomyomas. While the mean gray value of the standard deviation of leiomyomas was significantly lower than that of GISTs (20.6 ± 7.0 vs. 39.8 ± 9.3, *t* = 23.7, *P* < 0.0001), this indicates that the echo consistency of leiomyomas was more uniform than that of GISTs (Table 5). GISTs are potentially malignant tumors. The grayscale had a sensitivity, specificity, positive predictive value, and negative predictive value of 85.9%, 74.6%, 67.3%, and 89.7% for GIST diagnosis. Therefore, if the mean gray value is close to 58.9 ± 8.3 and the mean gray value of the standard deviation is close to 39.8 ± 9.3, it could be considered GIST. In contrast, if it is close to 39.5 ± 5.9 and the mean gray value of the standard deviation is close to 20.6 ± 7.0, it should be treated as leiomyoma. However, pathological studies remain the gold standard for diagnosis.

### 3.4. Lesions Undetermined by EUS before Endoscopic Resection

A total of 47 patients with upper gastrointestinal SELs (diffused infiltrated) were undetermined by EUS before endoscopic resection. The majority of lesions originated from the submucosa (51.1%, 24/47), muscularis mucosa (23.4%, 11/47), muscularis propria (12.8%, 6/47), and unclear origin (12.8%, 6/47). These lesions were found in the antrum (18/47), duodenum (2/47), gastric fundus (13/47), cardiac (3/47), gastric body (3/47), and esophagus (8/47). Furthermore, 31.9% (15/47) were pathologically determined chronic inflammation after endoscopic resection (Table 6).

## 4. Discussion

Subepithelial lesions of the gastrointestinal tract are defined as elevated lesions or bulge within the lumen that is usually covered by normal-appearing mucosa [2]. These tumors are characterized as an intramural growth, which cannot be fully determined either by standard luminal endoscopy or by barium contrast radiography [10]. Early endoscopic identification is crucial. Endoscopic ultrasound is the second phase in the assessment of SELs which provides valuable information to guide further management [1]. Moreover, EUS is the diagnostic investigation of choice to differentiate between intramural and extramural lesions and to assess the size, margins, origin layer, lesion echotexture and presence of adjacent lymph nodes, surrounding structures and is significantly more effective than endoscopy, transparietal ultrasonography, and CT scan [16–19]. It is important to recognize the normal five layers of the gastrointestinal wall in order to diagnose SELs and precisely achieve T-staging. The great advantage of EUS is the precise delineation of gastrointestinal wall layers which allows a detailed examination of the submucosal tumor morphology and the exact localization of the layer of origin [6]. Subepithelial lesions can be classified based on their location and precise echogenicity within the gastrointestinal wall layer. For example, cysts which are anechoic lesions within the submucosa, leiomyoma or GIST which are hypoechoic lesions emerging from the muscularis mucosa or propria [15]. Based on the EUS review, a decision can be made to decide between no further investigations, follow-up with EUS, or additional diagnostic or therapeutic strategy with resection when the lesion is suspected to be malignant [1, 20]. In recent years, a real-time contrast-enhanced EUS based on contrast-specific harmonic imaging modes and EUS elastography have become available to accurately discriminate between leiomyoma and GIST [21]. A study by Ignee et al. [21] reported avascular areas and hyperenhancement in a high percentage of GISTs, while leiomyoma consistently demonstrated hypoenhancement using contrast-enhanced EUS, suggesting that contrast-enhanced EUS is an appropriate method for distinguishing both entities. According to a study by Kamata et al. [22] on contrast-enhanced harmonic EUS, the majority of GISTs (49 of 58) presented with hyperenhancement, while benign submucosal tumors (4 of 15) demonstrated hyperenhancement. 21 of 58 GISTs showed inhomogeneous contrast enhancement, whereas 2 of 15 benign submucosal tumors showed inhomogeneous contrast enhancement. However, in lesions of less than 2 cm, hyperenhancement was found to be a more sensitive indicator of GISTs than inhomogeneous enhancement [22]. Moreover, contrast-enhanced harmonic EUS appeared to be useful for differential diagnosis and risk stratification of submucosal tumors [23].

**Figure 2:**
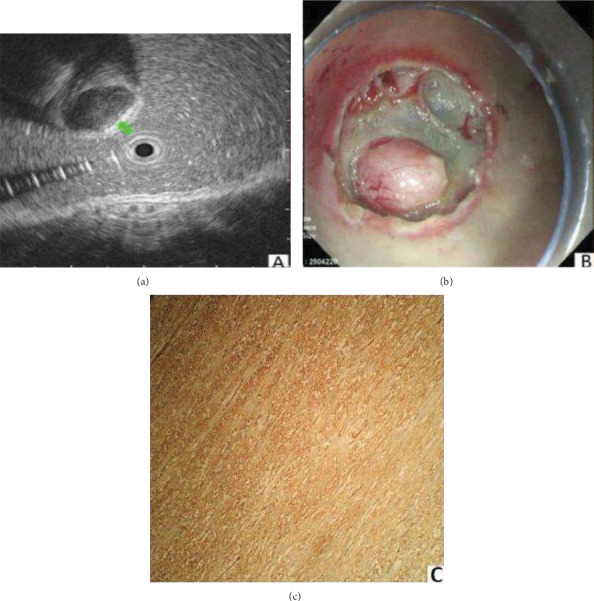
Leiomyoma was shown as a round homogenous hyperechoic tissue (a), the layer of the origin muscularis propria (b) and smooth muscle actin (SMA)(+), CD117(-), and CD34(-) (c).

**Figure 3:**
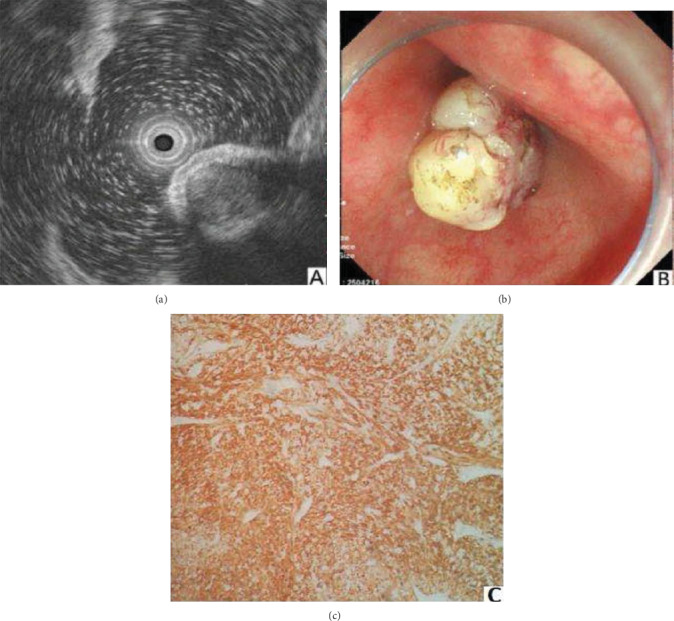
GIST was demonstrated as a round, uniform hyperechoic lesion of muscularis propria (a, b) and CD117(+) or CD34(+) and SMA(-) (c).

In our study, leiomyomas and GISTs consistent with their postoperative pathology were analyzed retrospectively. However, it is still hard to distinguish lesions with a small diameter. In this study, the esophageal leiomyomas were presented as homogenous hypoechoic masses from the second and fourth layers, with a regular and well-defined outline (Figure 2). According to Punpale et al. [24], esophageal leiomyomas are the most common benign tumors of the esophagus, as shown in our study. In contrast, GISTs were most commonly found in the stomach (Figure 3). A study by Guo et al. [11] indicated that small GISTs usually appear to be hypoechoic with a regular outline, while larger ones may have irregular outlines and inhomogeneous internal echoes (hyperechoic foci, cystic structures, etc.).

In this retrospective study, the diagnostic consistency of EUS in predicting the location of lesions was 88.2%. The presumptive diagnostic consistency of EUS with postoperative pathology was 80.1% (530/662), which was approximate to the previously reported rate (73%) [25]. The image density of EUS was used to distinguish the location of the lesion. The EUS showed better results, mainly for lipoma and ectopic pancreas (97.0%, 96.8%). However, GISTs showed poor results (63.0%), especially for lesions < 2 cm in diameters.

According to the National Comprehensive Cancer Network, if the GIST > 2 or < 2 cm with symptoms or < 2 cm with high-risk EUS characteristics should be removed [2, 26]. In 2013, Japan Gastroenterological Endoscopy Society recommended surgery for SETs < 2 cm suggestive of malignancy (an irregular border or a tumorous ulcer) on endoscopy. The European Society for Medical Oncology and the European Society of Gastrointestinal Endoscopy suggest that EUS should be performed 3 months after the detection of SETs < 2 cm in the esophagus, stomach, and duodenum, followed by an annual follow-up. If the lesions increase in size or became symptomatic, they should be removed [27].

In this study, the quantification of tumor tissue, echo intensity, and other parameters were measured by a grayscale histogram analysis, which showed that the mean gray value of GISTs was higher than that of leiomyomas. On the contrary, although GISTs had higher echo intensity, the echo uniformity of leiomyomas was more uniform than that of GISTs. Recently, Tuma et al. [28] reported that the measurement of ultrasound echo intensity by a grayscale histogram analysis could be used for differential diagnosis of focal renal lesions. Thus, EUS combined with a grayscale histogram analysis, or EUS itself with a gray value function, could be the gold standard method in future technologies for the identification and differentiation of leiomyoma and GIST.

In our study, 47 cases with upper gastrointestinal SELs were undetermined by EUS, and (6/47, 12.8%) showed unclear origin. It has been reported that the precise delineation of the depth of the tumor invasion sometimes obscured in the presence of glandular components, inflammatory infiltration, or scar [29, 30]. Furthermore, in postendoscopic resection, (15/47, 31.9%), SELs were pathologically characterized by chronic inflammation with diffuse gastrointestinal wall thickening. This phenomenon may be correlated with inflammatory exudation and explosion. The submucosal structure of these lesions was relatively slack; it was difficult to diagnose the lesions with irregular mixed echo or inhomogeneity. The accuracy of EUS can be influenced by ulcers [31, 32], histopathological changes, and large tumor size (>2 cm) [33, 34]. The quality of EUS imaging significantly reduces diagnostic accuracy [31]. In addition, the EUS-guided FNA/EUS-FNB procedures [2, 5, 32] and the grayscale histogram or the Fuzzy Inference analysis can be used for the diagnosis of submucosal tumors. In the case of undetermined SELs, endoscopists should pay more attention to chronic inflammation; finally, every effort should be made to improve the quality of EUS imaging.

This study has certain limitations which may provide opportunities for future research. First, this was a retrospective study of a computerized databank. The database was accurately managed prospectively to collect data for clinical research. Second, the study sample size was limited to a single center. Third, our main focus of findings was applied to differentiate the gray value of leiomyomas and GISTs in the upper gastrointestinal SELs. Fourth, prospective multicenter studies including more patients are required to validate the potential of the EUS combined with the grayscale histogram analysis in differentiating all types of upper gastrointestinal SELs.

In conclusion, the diagnostic reliability of EUS was high, indicating that EUS should be the first-line method for assessing SELs in the upper gastrointestinal tract. The grayscale histogram analysis of both leiomyomas and GISTs showed a significant difference between the mean gray value and the mean gray value of the standard deviation. The echo intensity of GISTs was higher than that of leiomyomas, but the echo-uniformity of leiomyomas was greater than that of GISTs. Thus, EUS combined with the grayscale histogram analysis is an effective method for the differentiation of leiomyomas and GISTs. Further multicenter research on the combination of EUS and grayscale analysis is needed to improve routine clinical practice.

## Figures and Tables

**Table 1:** Clinical characteristics of patients with SELs.

Characteristics	Cases (no = 662)
Sex	
Male	282
Female	427
SELs	
GIST	281
Leiomyoma	317
Ectopic pancreas	31
Lipoma	33
Age	
GIST	57.1±9.8
Leiomyoma	52.8±10.9
Ectopic pancreas	50.3±11.2
Lipoma	50.1±12.9
Tumor size (cm)	
GIST	1.5±0.9
Leiomyoma	1.2±0.6
Ectopic pancreas	1.9±0.9
Lipoma	2.1±0.9
Tumor location	
Esophagus	279
Stomach	378
Duodenum	5
Layer of origin	
Muscularis mucosa	242
Submucosa	69
Muscularis propria	350
Unclear	1

SELs: subepithelial lesions; GISTs: gastrointestinal stromal tumors; cm: centimeter.

**Table 2:** Diagnostic consistency of EUS before endoscopic resection.

Diagnosis of EUS	Esophagus	Stomach	Duodenum
GISTs	19	260	2
Leiomyoma	257	60	0
Ectopic pancreas	0	31	0
Lipoma	3	27	3
Total	279	378	5

EUS: endoscopic ultrasonography; GISTs: gastrointestinal stromal tumors.

**Table 3:** EUS consistency in predicting the location of lesions.

Depth of lesions	EUS	Endoscopic resection	Consistency (%)
Muscularis mucosa	242	185	76.4
Submucosa	69	61	88.4
Muscularis propria	350	338	96.6
Unclear	1	0	0
Total	662	584	88.2

EUS; endoscopic ultrasonography.

**Table 4:** Diagnostic consistency of EUS with pathology.

Diagnosis	EUS	Pathology	Consistency (%)
GISTs	281	177	63.0
Leiomyoma	317	291	91.8
Ectopic pancreas	31	30	96.8
Lipoma	33	32	97.0
Total	662	530	80.1

EUS: endoscopic ultrasonography; GISTs: gastrointestinal stromal tumors.

**Table 5:** Grayscale histogram analysis of GIST and leiomyoma.

	The mean gray value	The mean gray value standard deviation
GIST	58.9 ± 8.3^∗^	39.8 ± 9.3^∗^
Leiomyoma	39.5 ± 5.9^∗^	20.6 ± 7.0^∗^
*T*	57.0	23.7
*P*	*P* < 0.0001	*P* < 0.0001

^∗^The data were expressed as mean ± SD. There was a significant difference between GIST and leiomyoma in ^∗^the mean gray value (*t* = 57.0, *P* < 0.0001) and the mean gray value standard deviation (*t* = 23.7, *P* < 0.0001).

**Table 6:** Lesions undetermined by EUS.

Unclear (6)	Gastric antrum	Chronic inflammation (2)	Ectopic pancreas (1)	
	Gastric fundus	Chronic inflammation (2)	GIST (1)	

Submucosa (24)	Esophagus	Leiomyoma (2)	GIST (2)	Cyst (1)
	Gastric antrum	Chronic inflammation (3)	Ectopic pancreas (2)	
		Lymphoma (1)	Neuroendocrine tumor (1)	Polyps (1)
		Fibromyxoma (1)	Adenomyoma (1)	Dieulafoy's lesion (1)
	Gastric fundus	Chronic inflammation (1)	GIST (1)	Lipoma (1)
		Vascular malformations (1)		
	Cardiac	Chronic inflammation (1)		
	Gastric body	Ectopic pancreas (1)	Lipoma (1)	
	Duodenum	Ectopic pancreas (1)		

Muscularis mucosa (11)	Esophagus	Leiomyoma (3)		
	Gastric antrum	Ectopic pancreas (2)		
	Gastric fundus	Chronic inflammation(4)		
	Cardiac	Leiomyoma (1)		
	Duodenum	Neuroendocrine tumor (1)		

Muscularis propria (6)	Gastric antrum	Leiomyoma (1)	Chronic inflammation (1)	
	Gastric fundus	Lymphoma (1)	Fibromyxoma (1)	
	Cardiac	Chronic inflammation (1)		
	Gastric body	Leiomyoma (1)		

EUS: endoscopic ultrasonography; GISTs: gastrointestinal stromal tumors.

## Data Availability

All data relevant to the study are included in the article or uploaded as supplementary.

## Abbreviations

EUS: Endoscopic ultrasonography
SELs: Subepithelial lesions
GISTs: Astrointestinal stromal tumors
EMR: Endoscopic mucosal resection
ETR: Endoscopic trepanned resection
STER: Submucosal tunneling endoscopic resection
EFTR: Endoscopic full-thickness resection
ESD: Endoscopic submucosal dissection
CT: Computed tomography
cm: Centimeter.
